# Functions and Mechanisms of the Voltage-Gated Proton Channel Hv1 in Brain and Spinal Cord Injury

**DOI:** 10.3389/fncel.2021.662971

**Published:** 2021-04-09

**Authors:** Junyun He, Rodney M. Ritzel, Junfang Wu

**Affiliations:** ^1^Department of Anesthesiology and Center for Shock, Trauma and Anesthesiology Research (STAR), University of Maryland School of Medicine, Baltimore, MD, United States; ^2^University of Maryland Center to Advance Chronic Pain Research, University of Maryland, Baltimore, MD, United States

**Keywords:** voltage-gated proton channel Hv1, NOX2, acidification, ROS, microglia, traumatic brain injury, spinal cord injury, stroke

## Abstract

The voltage-gated proton channel Hv1 is a newly discovered ion channel that is highly conserved among species. It is known that Hv1 is not only expressed in peripheral immune cells but also one of the major ion channels expressed in tissue-resident microglia of the central nervous systems (CNS). One key role for Hv1 is its interaction with NADPH oxidase 2 (NOX2) to regulate reactive oxygen species (ROS) and cytosolic pH. Emerging data suggest that excessive ROS production increases and requires proton currents through Hv1 in the injured CNS, and manipulations that ablate Hv1 expression or induce loss of function may provide neuroprotection in CNS injury models including stroke, traumatic brain injury, and spinal cord injury. Recent data demonstrating microglial Hv1-mediated signaling in the pathophysiology of the CNS injury further supports the idea that Hv1 channel may function as a key mechanism in posttraumatic neuroinflammation and neurodegeneration. In this review, we summarize the main findings of Hv1, including its expression pattern, cellular mechanism, role in aging, and animal models of CNS injury and disease pathology. We also discuss the potential of Hv1 as a therapeutic target for CNS injury.

## Introduction

The central nervous system (CNS) injuries or diseases such as stroke, traumatic brain injury (TBI), spinal cord injury (SCI), and multiple sclerosis (MS), are the leading causes of death and disability in the United States and around the world. To date, no neuroprotective or neurorestorative therapy has clearly improved long-term recovery. This may reflect, in part, an incomplete understanding of the complex pathobiological mechanisms involved. Numerous experimental observations including ours (Faden et al., 2016; Skovira et al., 2016; Li Y. et al., 2020; Ritzel et al., 2020) indicate that persistent neuroinflammation contributes to long-term neurological functional deficits. However, no preferred current treatments specifically target neuroinflammatory mechanisms for CNS damage. Thus, identifying new targets to effectively reduce CNS injury-induced neuroinflammation remains an important research direction.

As the major cellular component of the innate immune system in the CNS, microglia play a critical role in neuroinflammation following CNS trauma (Loane and Byrnes, 2010). In response to injury, microglia can produce neuroprotective factors, clear cellular debris and orchestrate neurorestorative processes that are beneficial for neurological recovery. However, dysregulated microglia can also produce high levels of pro-inflammatory and cytotoxic mediators that can injure cells and hinder CNS repair (Loane and Kumar, 2016). Theoretically, resident microglia should work in concert to fine-tune inflammatory responses, scavenge debris, and promote remodeling and repair after injury, thereby contributing to successful wound healing. However, microglia often exhibit a sustained neurotoxic phenotype continuing for months to years after CNS trauma, associated with progressive neurodegeneration and related neurological dysfunction (Faden et al., 2016).

Proton (H^+^)-sensitive channel and exchanger in microglia include the hydrogen voltage gated channel 1 (HVCN1) and Na^+^/H^+^ exchanger (NHE). The HVCN1 gene encodes a voltage-gated protein channel protein (Hv1/VSOP) that is primarily expressed in phagocytes in the immune system (Ramsey et al., 2006; Sasaki et al., 2006) and is one of the major ion channels expressed in CNS-resident microglia (Wu et al., 2012). It is reported that Hv1 is not expressed by neurons or astrocytes in the mouse brain (Wu et al., 2012). In contrast, NHE1 is ubiquitously expressed in all cell types in the CNS. While NHE1 is important for maintaining the basal intracellular pH (pHi) in microglia, cell migration, and calcium triggering, Hv1 mediates pH regulation and membrane hyperpolarization in microglia (Luo et al., 2021). Although the voltage-dependent proton current was first reported forty years ago (Thomas and Meech, 1982), its gene and molecular structure were not identified until 2006 (Ramsey et al., 2006; Sasaki et al., 2006). The crystal structure is a chimeric construct composed of the parts of 3 different proteins and forms a trimer, in contrast to the dimer-forming Hv1 channel (Takeshita et al., 2014). To date, one key physiological function of Hv1 is the regulation of NADPH oxidase (NOX) activity in phagocytes (Ramsey et al., 2009). Under pathological conditions, microglial Hv1 is required for NOX-dependent generation of reactive oxygen species (ROS) by providing charge compensation for exported electrons via proton extrusion, thereby relieving the intracellular acidosis that occurs following respiratory burst. Excessive NOX/ROS expression and tissue acidosis can damage neurons and glia, and participates in the pathophysiology of neuroinflammatory disorders (von Leden et al., 2017). However, Hv1 also functions independently of the NOX/ROS pathway, for example in snail neurons, basophils, osteoclasts, and cancer cells (Seredenina et al., 2015). Early work from experimental stroke models using transgenic knockout (KO) mice provides a strong rationale for Hv1 as a potential therapeutic target for the treatment of ischemic brain injury (Wu et al., 2012).

Generally, ion channels including Hv1 are potent drug targets (Seredenina et al., 2015). To facilitate the development of clinically useful Hv1-active drugs, this review summarizes the current research on the function and mechanisms of the Hv1 channel in CNS injury models including various ischemic and traumatic brain injuries, neurodegenerative disease, and spinal cord injury. We also discuss recent studies on disrupted Hv1 in age-related pathologies, highlighting its involvement in chronic CNS injury and neurodegenerative diseases. The temporal and spatial expression of Hv1 channel are reviewed in CNS resident cells as well as on injury-responsive immune cells. Potential cellular mechanism studies addressing the role of Hv1 in CNS injury-mediated pathobiology are highlighted, including microglial Hv1-triggered oxidative stress and neuroinflammation. Lastly, we review recent novel mechanistic discoveries on Hv1-driven intracellular and extracellular acidosis in injured brain and spinal cord tissues, and provide therapeutic options, with an emphasis on the depletion or inhibition of the Hv1 channel for the prevention of acidotoxicity, oxidative stress damage, and inflammatory neurodegeneration after CNS trauma.

## The Discovery and Structure of the Voltage-Gated Proton Channel Hv1

**FIGURE 1:**
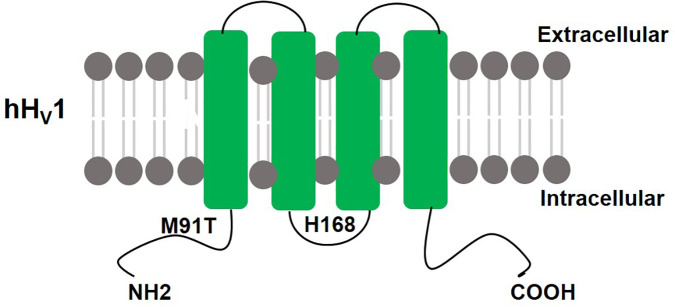
Schematic structure of hHv1: hHv1 has four transmembrane domains with N terminus and C terminus lie inside the cells. H168 is crucial for ΔpH-dependent gating, M91T mutant requires approximately 0.5 pH units more alkaline pH values than wild-type Hv1 to activate proton extrusion.

The voltage-dependent proton current was first reported in neurons of the land snail *Helix aspersa* (Thomas and Meech, 1982). Decades later in 2006 the Hv1 gene was identified simultaneously by two groups in both *Mus musculus* (Sasaki et al., 2006) and *Homo Sapiens* (Ramsey et al., 2006). The human Hv1 (hHv1) gene is located on the chromosome 12 at q24.11 and has seven exons. The hHv1 protein is predicted to be 273 amino acids in length (31.7 kD, pI = 6.62) and forms four transmembrane (TM) segments (s1–s4) (see Figure 1). The TM structure and placement of charged residues in hydrophobic domains is conserved among the vertebrate Hv1 orthologs. The N-terminal of Hv1 contains a voltage-sensitive domain (VSD) but is not conserved in sequence. The VSD of Hv1 is unique due to the lack of pore domain which is totally different from the other voltage-gated ion channels, and therefore Sasaki et al. (2006) first named it voltage sensor domain only protein (VSOP). The VSD has dual roles of voltage-sensing and proton permeation. Its gating is sensitive to pH and Zn^2+^ (Ramsey et al., 2006). H^168^ is crucial for ΔpH-dependent gating of the hHv1 (Cherny et al., 2018). The crystal structure of mouse Hv1 was presented in the resting state at 3.45Å resolution, providing a platform for understanding the general principles of voltage-sensing and proton permeation (Takeshita et al., 2014).

There are two isoforms of Hv1, long and truncated. HVCN1S is a short isoform lacking the first 20 amino acids via alternative mRNA splicing (Hondares et al., 2014). It is specifically expressed at higher levels in chronic lymphocytic leukemia (CLL) cells and other B cell lines than is the longer isoform, but only weakly expressed in normal B cells. HVCN1S mediates stronger currents upon PKC phosphorylation and increases BCR signaling, proliferation, and chemokine-dependent migration, which together confer a growth advantage to malignant B cells and contribute to the disease pathogenesis of CLL (Hondares et al., 2014).

In human sperm, Hv1 is post-translationally cleaved to a N-terminus truncated form that lacks the first 68 amino acids, generating Hv1Sper which has a molecular weight of 25 kDa (Berger et al., 2017). The pH-control of Hv1Sper and Hv1 is distinctively different. Voltage gating of Hv1 is intimately coupled to ΔpH across the cell membrane regardless of the changes in the intracellular and extracellular pH. While in Hv1Sper, gating is sensitive not only to ΔpH, but also to the changes in intracellular and extracellular pH. As a result, Hv1 can only export protons and alkalize sperm, whereas Hv1Sper can either alkalize or acidify sperm, depending on the pH of the environment (Berger et al., 2017).

A missense mutation of Hv1 at the 91 site from methionine to threonine (M91T) in epithelial cells requires approximately 0.5 pH units more alkaline mucosal pH values than wild-type Hv1 to activate proton extrusion, providing both functional and molecular indication that Hv1 mediates pH-regulated acid secretion by the airway epithelium (Iovannisci et al., 2010).

## The Temporal and Spatial Expression of Hv1 in the Cns

The Hv1 channel is a membrane protein that is highly expressed in cells of the immune system including eosinophils (DeCoursey et al., 2001), neutrophils (DeCoursey et al., 2000), basophils (Musset et al., 2008), B cells (Capasso et al., 2010), T cells (Schilling et al., 2002), monocytes (Musset et al., 2012a), macrophages (Kapus et al., 1993), dendritic cells (Szteyn et al., 2012), and mast cells (Kuno et al., 1997), suggesting that Hv1-driven signaling may be involved in a variety of immunological processes. In the CNS, Hv1 mRNA expression patterns in rat primary cultured neurons, microglia, and astrocytes revealed that its expression is predominantly limited to microglia (Li et al., 2021). The basal level of Hv1 in cultured astrocytes and neurons was extremely low or undetectable (Wu et al., 2012; Li et al., 2021). In the healthy mouse brain, Hv1 was found to be exclusively expressed in microglia, but not in neurons or astrocytes (Wu et al., 2012). The expression level of Hv1 in the mouse CNS is developmentally increased, i.e., non-detectable in the neonatal stage, detectable in adulthood by western blot, and 2–3-fold higher in the aged brain compared with that in the adult brain (Wu, 2014; Zhang et al., 2020). This likely reflects the increased activation state of microglia that occurs with normal aging (Harry, 2013; Norden and Godbout, 2013).

In response to CNS insults, increased Hv1 protein expression was observed in whole brain lysates at 24 h after permanent middle cerebral artery occlusion (MCAO) (Wu et al., 2012). In a mouse moderate/severe brain injury using a controlled cortical impact (CCI) model of experimental TBI, Hv1 mRNA and protein expression were remarkably increased at 24 h post-injury and remained elevated for up to 4 weeks in the ipsilateral cortex and hippocampus (Ritzel et al., 2021). qPCR analysis revealed a virtual absence of Hv1 mRNA expression in the microglia-depleted brain (Ritzel et al., 2021), further confirming that expression of this channel is largely restricted to microglia within the CNS. Dysregulation of Hv1 expression in the injured spinal cord after moderate/severe contusion in mice was also recently reported (Li et al., 2021). qPCR analysis showed a rapid up-regulation (40-fold) of Hv1 mRNA, starting at 3 days post-injury and continuing for up to 28 days in both male and female mice. This was confirmed in CD11b^+^ microglia/macrophages isolated from the adult spinal cord at 3 days post-injury. *In situ*, cells immunoreactive for Hv1 coexpress ionized calcium-binding adaptor molecule 1 (Iba1), a known marker for microglia/macrophages (Li et al., 2020b). Furthermore, a nearly four-fold increase in Hv1 protein expression was observed in spinal cord tissue at 3 days post-injury (Li et al., 2021). These results demonstrate that the expression level of the Hv1 channel is dysregulated following CNS injury.

## Cellular Mechanism and Physiological Function of Hv1 Channels

### NOX2-Mediated ROS Signaling Pathways

The best-described function of Hv1 is the regulation of NOX2 activity in phagocytes. During respiratory burst, electrons from NADPH are transferred outside of the cell, leading to membrane depolarization; at the same time, protons are accumulated in the cytoplasm, leading to acidification of the cytosol (El Chemaly et al., 2010). NOX activity, depolarization, and intracellular acidification are key stimuli to activate Hv1. Both membrane depolarization and cytosol acidification inhibit the activity of NOX2, which can be reversed by Hv1-mediated extrusion of protons from the cytoplasm, thereby maintaining physiological membrane potential and re-establishing normal pH. Inhibition of Hv1 activity would thus portend a decrease in proton extrusion that could lead to intracellular acidosis. For example, in Hv1-deficient neutrophils, the cytosolic pH is more acidic than in wild-type (WT) cells following stimulation with phorbol myristate acetate (PMA) (El Chemaly et al., 2010). In addition to superoxide production, Hv1 also sustains calcium entry and migratory capacity (El Chemaly et al., 2010).

Hv1-deficient eosinophils have also been shown to produce significantly less ROS upon PMA stimulation compared with WT eosinophils (Zhu et al., 2013). However, Hv1-depleted eosinophils did not show an impairment in calcium mobilization or migration ability as demonstrated in neutrophils. Interestingly, Hv1-deficient eosinophils underwent significantly greater cell death after PMA stimulation, suggesting that intracellular acidosis during respiratory burst may be more severe in this cell type (Zhu et al., 2013).

In adaptive immune cells, Hv1 modulates B cell antigen receptor (BCR) signal strength in B cells (Capasso et al., 2010). Generation of ROS was lower in Hv1-deficient B cells, resulting in attenuated BCR signaling via impaired BCR-dependent oxidation of Src homology region 2 domain-containing phosphatase-1 (SHP-1). Hv1 KO mice showed reduced activation of the spleen tyrosine kinase (SYK) and protein kinase B (PKB, also known as Akt) signaling pathways, impaired mitochondrial respiration and glycolysis, and diminished antibody responses (Capasso et al., 2010). While little is known regarding the role of Hv1 in T cells, there is evidence that ablating Hv1 in Jurkat T cells induces acidification which promotes apoptotic cell death (Asuaje et al., 2017). It remains to be seen whether Hv1 plays a similar role in modulating T cell receptor (TCR) signaling pathways and downstream effector functions.

In microglia, Hv1 channels were reported to conduct protons to the extracellular space as charge compensation and to help sustain ROS production by NOX2 (Wu et al., 2012). However, Kawai et al. (2017, 2018) observed that Hv1-deficiency enhanced the extracellular ROS signal in primary cultured microglia, as well as in Kupffer cells (Kawai et al., 2020). This may be apparently paradoxical considering numerous evidence showing that Hv1 supports ROS generation, but still sheds light on the complex regulatory mechanism of Hv1 in context of diverse physiological states of microglia.

### Non-NOX Dependent Hv1 Function in ROS Production

Within the medullary thick ascending limb, Hv1 is found to localize in the mitochondria membrane where it modulates the formation of ROS by complex I on the mitochondrial membrane rather than by NOX-mediated ROS production. It points to a novel pathway of Hv1 independent of its traditional role in maintaining cell membrane potential and intracellular pH (Patel et al., 2019).

### pH Regulation

It is noteworthy that in many instances Hv1 functions independently of NOX. As a proton channel, Hv1 is responsible for conducting protons and thus regulating the intracellular or extracellular pH. For example, in human spermatozoa, Hv1 is highly expressed in the flagellum. Activated Hv1 allows outward conduction of protons, resulting in intracellular alkalization and activation of spermatozoa (Lishko et al., 2010). Hv1 activity has been suggested to play a role in a number of functions in sperm, including motility, capacitation, sperm-zona pellucida interaction, acrosome reaction, and sperm-oocyte fusion (Lishko et al., 2010; Musset et al., 2012b; Zhao et al., 2018; Yeste et al., 2020).

In osteoclast cells, Hv1 helps release H^+^ ions to acidify the extracellular milieu and dissolve mineralized bone, promoting bone resorption (Nordstrom et al., 1995). In airway epithelia, Hv1 is involved in the maintenance of the physiological pH of the airway and lung environment (Fischer et al., 2002; Cho et al., 2009; Iovannisci et al., 2010; Fischer, 2012). Hv1 activation by PMA or anti-IgE in basophils may cause histamine release, which is coupled with the ability of Hv1 to maintain cytosolic pH but not linked with NOX2 due to its lack of expression in basophils (Musset et al., 2008). This same mechanism of regulating histamine release is also found in mast cells (Marone et al., 1986).

In addition to regulating the intracellular pH, Hv1 is also responsible for regulating the pH of phagocytic vacuole of neutrophils. In human cells, the vacuolar pH is around 9. By contrast, in Hv1 KO mouse neutrophils, the vacuolar pH rose above 11, and the cytosol acidified excessively, demonstrating that Hv1 plays an important role in charge compensation (Levine et al., 2015). Furthermore, Hv1 is required for proper endosomal acidification and subsequent toll-like receptor 9 (TLR9) signaling in plasmacytoid dendritic cells (Montes-Cobos et al., 2020). Hv1 deficiency delayed endosomal acidification, consequently limiting protease activity and the secretion of type I interferons (IFN-I). Whether Hv1 impacts antigen presentation by dendritic cells in the context of microbial infection remains to be seen.

## Pathological Function of Hv1 Channels

### Hv1 in Brain Aging and Age-Related Pathology

Aging is a major risk factor for many human pathologies, including metabolic, inflammatory, and neurodegenerative conditions. The role of ROS and Ca^2+^ in aging and age-related disease has been extensively reviewed (Madreiter-Sokolowski et al., 2020). Each have been strongly implicated in the development and progression of age-related pathologies. By ensuring Ca^2+^ entry through charge compensation, the Hv1 proton channel helps to sustain NOX2-induced ROS production and other phagocyte effector functions critical for innate immunity (El Chemaly et al., 2010). Age-associated defects in neutrophil migration, adhesion, and oxidative burst activity have been documented in young Hv1-deficient mice (Ramsey et al., 2009; El Chemaly et al., 2010; Capasso et al., 2011; Okochi et al., 2020). These observations have led many to examine the role of Hv1 in the context of aging. Given its role in rebalancing pH and charges across the plasma membrane, dysregulated Hv1 activity could provide both a mechanism of age-related disease and a therapeutic target. Although few studies have examined the relationship between Hv1 and innate immune function in naturally aging mice, emerging data suggest that Hv1 activity may play a key role in age-dependent responses to injury.

Studies investigating Hv1 expression across the lifespan have identified age- and region-dependent increases in the brain. The highest expression level was seen in the striatum of WT mice and was associated with increased expression of markers of microglia (e.g., CD11b, CX3CR1, and CD68) (Kawai et al., 2021). These findings suggest that Hv1 may be involved in the transition of microglia from a ramified phenotype to an ameboid morphology indicative of the heightened activation state seen with advanced age (Kawai et al., 2021). Interestingly, despite showing preferential expression in the striatum, no functional significance could be detected in this, or any other, region of the brain in young KO mice. However, the authors found that morphological features of microglia activation and differential gene expression in the cortex were significantly increased in aged KO mice. While this study highlights a role for Hv1 in age-related microglia activation and chronic neuroinflammation, it is important to point out that the KO mice at all ages showed no severe neurological impairment. It remains to be seen whether these age-related changes are adaptive, if Hv1 activity itself is increased in the same region-dependent manner, and which environmental factors are responsible for promoting its expression.

Inflammation subsequent to microglia activation has been demonstrated to be a key driver of age-related cognitive deficits (Udeochu et al., 2016). Due to its high expression in microglia, it is not unreasonable to assume Hv1 could play a detrimental role in neurological decline. However, presently there is no data to support this notion. A study assessing neuroinflammation after postoperative surgery found that Hv1 protein expression was significantly increased in the hippocampus of 18 months old WT mice, but the functional importance of this change with relation to cognition remains to be seen (Zhang et al., 2020). One possible consequence of increased Hv1 activity includes higher production of ROS and oxidative stress damage. Recent work has demonstrated a concomitant increase in NOX2 and ROS levels within the aging hippocampus (Fan et al., 2019). Indeed, recent evidence suggests that Hv1 may promote transcription of oxidative stress genes (e.g., Nrf2, Sod2, and Gpx1) in an age-dependent manner (Kawai et al., 2017). Utilizing global KO mice of various ages, the authors demonstrated that functional Hv1 is increasingly more important during early middle age (i.e., 6 months), when its loss-of-function appears to coincide with higher expression of oxidative stress genes. That deletion of a gene partially required for NOX2-mediated ROS production would result in increased oxidative stress seems paradoxical unless one is to consider the possibility that it removes a brake on, or is overcompensated by increased expression of, Hv1-independent drivers of ROS production. In the same study it was also reported that neuroprotection to experimental stroke was seen in old, but not young mice. While these results are surprising, it supports an adaptive role for Hv1 in age-related responses to brain injury. Consistent with notion, microarray profiling revealed higher expression of Hv1 and other oxidative stress genes in the ischemic hemisphere of old mice compared to young mice (Sieber et al., 2014). The immunological response to stroke is dramatically altered with age, with older mice showing significantly greater neutrophil infiltration and higher rates of hemorrhagic transformation (Ritzel et al., 2018). It is possible that the age-dependent protection seen in older KO mice is due to decreased transmigration and reactivity of neutrophils in the ischemic brain, rather than microglia-driven. Nevertheless, neuroprotection in young KO mice has been documented in models of traumatic CNS injury, hypoperfusion-induced white matter injury, and multiple sclerosis (Li et al., 2021; Ritzel et al., 2021). Moreover, our group (Ritzel et al., 2021) showed that protection persisted for weeks and months following TBI. These findings indicate there could be important interactions between age, Hv1, and the onset, duration, and type of injury.

It is worth reiterating that Hv1 expression is not exclusive to phagocytes of myeloid lineage, as some groups have reported expression in B and T lymphocytes (Fernandez et al., 2016). In B cells, Hv1 has been shown to regulate the strength of BCR signaling (Capasso et al., 2010). Despite the well characterized effects of aging on B cells and autoimmunity, few studies have examined the age-dependency of Hv1 function in adaptive immunity (Ma et al., 2019). One report indicates Hv1 KO mice develop an autoimmune disorder phenotype with advanced age (Sasaki et al., 2013). This was characterized by splenomegaly at 6 months of age, higher CD44 expression on T cells, and increased production and deposition of autoantibodies in the kidney coincident with nephritis. If confirmed, these findings suggest that Hv1 may play a more important role in the adaptive arm of the immune system than previously appreciated. However, these results should be interpreted with caution, as it has been suggested that these age-associated phenotypic features may be dependent on external and intrinsic factors such as housing conditions and C57Bl/6 substrains, respectively (Capasso, 2014).

One major caveat to these studies is the over interpretation of results generated using germline KO mice, which may have hidden developmental aberrations that affect the context of Hv1 activity, including the potential for other genes to compensate for this activity. For this reason, the advent of cell type-specific conditional gene deletion and temporal-controlled inducible gene deletion systems will prove indispensable for understanding how the aging process effects Hv1 activity, and by extension, the pathogenesis of age-related pathology. Furthermore, future studies using pharmacologic inhibitors to selectively target Hv1 should include aged animals to determine whether there is an enhanced therapeutic response. The established roles of ROS, ionic dysregulation, inflammation, and metabolism in aging and disease continue to provide sound rationale for establishing the underlying role of Hv1 in this process.

### Hv1 in Ischemic Stroke

A detrimental role for Hv1 in stroke was first reported in 2012 (Wu et al., 2012). By utilizing Hv1 KO mice and both transient and permanent MCAO ischemic stroke models, Wu and colleagues showed that mice constitutively lacking Hv1 had reduced infarct volumes and more favorable neurological deficit scores at 24 h after stroke independent of reperfusion. This protection was due to a reduction in ROS attributable to the interaction between microglial Hv1 and NOX2. The authors demonstrated this by generating bone marrow chimeric mice that lacked either microglial or bone marrow-derived Hv1. These data supported the notion that brain resident microglial Hv1 are the primary mediators for NOX/ROS-induced brain damage 1d after MCAO. However, during the first week of injury, large numbers of the blood-borne leukocytes entered the brain which highly express Hv1 (Schilling et al., 2003; Gelderblom et al., 2009). Thus, the contribution of peripheral Hv1 cannot be excluded in the post-acute stages of stroke.

In a photothrombotic stroke model, Hv1 KO mice displayed smaller brain infarction and fewer motor coordination deficits compared to the WT group during the first three days after injury (Tian et al., 2016), in part by reducing the pro-inflammatory (M1)- and increasing the anti-inflammatory (M2)-type polarization of microglia and macrophages (Tian et al., 2016). Furthermore, Kawai et al. (2017) reported the neuroprotective effects of Hv1 deficiency on infarct volume in experimental stoke occur in an age-dependent manner. ROS production was noted to be slightly decreased in Hv1 KO mice at younger stages of development (1 day, 5 days, 3 weeks old), but drastically increased in older KO mice (6 months old) compared with age-matched WT mice. Neuroprotection was seen in older Hv1 KO mice, whereas no protection was evident in the younger KO mice (9 weeks old) (Kawai et al., 2017). These findings were confirmed in Hv1 mutant Dahl salt-sensitive rats in both transient and permanent MCAO models. Although proton currents were large in mouse microglia and virtually absent in rat microglia (Wu et al., 2012), Hv1 KO rats exhibited less edema and bleeding in the brain, indicative of attenuated cerebrovascular injury (Li et al., 2019). Taken together, these results suggest that ablation of Hv1 is a promising therapeutic target for the treatment of ischemia stroke. The therapeutic effects of Hv1 deficiency in various animal models is summarized in Table 1. Limitations of these studies reflect the fact that transgenic mice do not relate directly to a therapeutic approach.

**TABLE 1:** Therapeutic effect of Hv1 deficiency (Hv1 knockout) in various animal models.

**CNS injury models**	**Targeted tissue**	**Cellular and functional outcomes**	**References**
Mouse transient or permanent MCAO	Brain	Changed ROS production and neuronal death, reduced infarct volume and increased neurological deficit scores	Wu et al., 2012; Kawai et al., 2017
Mouse photothrombotic stroke	Brain	Reduced ROS production, shifted microglial polarization from M1 to M2 state, reduced brain infarction and motor coordination deficits	Tian et al., 2016
Rat transient or permanent MCAO	Brain	Neuronal and vascular protection	Li et al., 2019
Mouse cuprizone-induced MS	Brain	Reduced ROS production and microglial activation, increased OPC proliferation and mature oligodendrocytes, reduced demyelination and improved motor function	Liu et al., 2015
Mouse MS by LPC injection into the corpus callosum	Brain	Reduced ROS and autophagy, and reduced myelin damage	Chen et al., 2020
Mouse hypoperfusion model of white matter injury by bilateral common carotid artery stenosis	Brain	Reduced ROS and pro-inflammatory cytokines production, shifted microglial polarization from M1 to M2 state, enhanced OPC proliferation and differentiation, attenuated the disruption of white matter integrity and improved working memory	Yu et al., 2020
Mouse traumatic brain injury	Brain	Reduced microglia proliferation and ROS production, limited tissue acidosis and intracellular acidosis, reduced chronic brain inflammation, improved neurological function	Ritzel et al., 2021
Mouse spinal cord injury	Spinal cord	Reduced tissue acidosis, NOX2/ROS production, decreased pro-inflammatory cytokine, microglia, leukocyte infiltration, and phagocytic oxidative burst, reduced reactive astrogliosis and CSPGs expression, reduced oligodendrocytes and neuronal cell death, reduced demyelinated areas, and cavity formation, and improved locomotor function	Li et al., 2020a,b, 2021; Murugan et al., 2020

*MCAO, middle cerebral artery occlusion; MS, multiple sclerosis; LPC, lysophosphatidylcholine; ROS, reactive oxygen species; M1, pro-inflammatory microglia; M2, anti-inflammatory microglia; NOX2, NADPH oxidase 2; OPC, oligodendrocyte progenitor cell; CSPGs, chondroitin sulfate proteoglycans.*

### Hv1 in CNS Demyelination

Multiple sclerosis (MS) is a chronic CNS disease characterized by autoimmune attack of the myelin sheath by T lymphocytes, resulting in inflammation and demyelination. In a cuprizone-induced mouse model of MS, Liu et al. (2015) showed that mice lacking Hv1 are partially protected from demyelination and motor deficits compared to WT mice. This effect was associated with reduced ROS production, attenuated microglial activation, increased oligodendrocyte progenitor cell proliferation, and increased numbers of mature oligodendrocytes, reaffirming the role of the Hv1 proton channel in controlling NOX-dependent ROS production in the pathogenesis of MS (Liu et al., 2015). Using an established two-point model of demyelination by injecting lysophosphatidylcholine (LPC) into the corpus callosum, a recent study identified that LPC-mediated myelin damage was reduced in Hv1 KO mice, evidenced by reduced ROS generation and autophagic activation in microglia (Chen et al., 2020). The link between Hv1 and autophagic function in microglia requires further investigation.

Oligodendrocyte progenitor cells (OPC) are believed to be the most fragile cells in hypoxia-ischemia-induced demyelination (Yu et al., 2018). To investigate the contribution of Hv1 to OPC damage, Yu et al. (2018) employed an *in vitro* model of co-culture with microglia. The authors found that, following oxygen-glucose deprivation, OPCs co-cultured with Hv1 KO microglia had attenuated apoptosis and greater proliferation and differentiation than those co-cultured with WT microglia. This protection was associated with decreased phosphorylation and activation of extracellular signal-regulated kinase (ERK) 1/2 and p38 mitogen-activated protein kinase (MAPK), and attenuated production of ROS and pro-inflammatory cytokines in microglia (Yu et al., 2018).

In a hypoperfusion model of white matter injury created by bilateral common carotid artery stenosis, genetic ablation of Hv1 attenuated the disruption of white matter integrity and improved working memory by enhancing OPC proliferation and differentiation into mature oligodendrocytes (Yu et al., 2020). Microglia-OPC co-cultures suggested that PI3K/Akt signaling was involved in Hv1 deficiency-induced anti-inflammatory-type microglial polarization and concomitant OPC differentiation. Thus, these results suggest that Hv1-NOX-ROS signaling contributes to the pathological changes in CNS demyelination.

### Hv1 in Traumatic Brain Injury and Spinal Cord Injury

**FIGURE 2:**
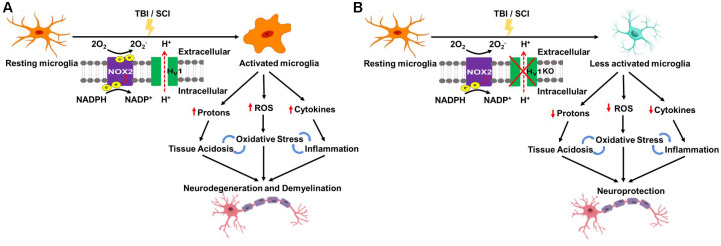
Schematic of Hv1 as a key driver of tissue acidosis, oxidative stress, and neuroinflammation in traumatic central nervous system (CNS) injury. **(A)** Traumatic brain injury (TBI) and spinal cord injury (SCI) lead to Hv1-driven microglia activation which causes tissue acidosis, oxidative stress, and an increase in proinflammatory cytokine levels. Consequently, there is an increase in neuronal loss, functional deficits, and poor long-term recovery. **(B)** Hv1 deficiency (KO) limits microglia activation and thereby rectifies trauma-induced reductions in environmental pH, abolishes ROS production, ameliorates neuroinflammation, and as a result, slows the progressive worsening of neuronal injury and functional deficits following SCI/TBI.

Although it is well known that Hv1 regulates intracellular pH, and aids in compensation for NOX-dependent generation of ROS, a direct connection between acidosis, ROS, and inflammation has not been established until recently. Work from our group has demonstrated that Hv1 is a key driver of tissue acidosis, oxidative stress, and neuroinflammation in models of traumatic brain injury and spinal cord injury (Li et al., 2021; Ritzel et al., 2021; see Figure 2).

Tissue acidosis in the CNS is a common occurrence in ischemic stroke and the brain/spinal cord trauma and a strong predictor of acute clinical function and long-term outcome (Marmarou, 1992; Marmarou et al., 1993; Clausen et al., 2005). The underlying mechanisms of pathological acidosis in CNS injury are not well understood, however, growing evidence suggests a positive association with inflammation. In a well-characterized CCI mouse model of experimental TBI, we investigated the cellular and molecular mechanisms of brain acidosis (Ritzel et al., 2021). We demonstrated that TBI causes intracellular and extracellular acidosis that persist for weeks after injury. Microglia proliferation and production of ROS were increased during the first week. When microglia were depleted by the colony stimulating factor 1 receptor (CSF1R) inhibitor, PLX5622, TBI-induced extracellular acidosis, oxidative stress, and inflammation were markedly decreased during the acute stages of injury. These data suggest that TBI-activated microglia participate in the regulation of pathological brain acidosis through a proton extrusion mechanism, indicating a direct connection between acidosis and inflammation. Moreover, we believe this may be a chronic process that contributes to chronic brain inflammation and progressive neurodegeneration. Using Hv1 KO mice, we demonstrated attenuated ROS production and increased intracellular acidosis in both microglia and brain-infiltrating myeloid cells during the acute period after TBI. Importantly, Hv1 KO mice exhibited lasting neuroprotection and improved functional recovery months after injury, suggesting therapeutic strategies to alleviate acidosis could have possible benefits even in the chronic stages of injury. Our data provide new cellular and molecular insights into the relationship between inflammation, acidosis, and head injury (Ritzel et al., 2021).

Using a contusion model of SCI in adult female mice, we demonstrated that depletion of Hv1 significantly attenuated tissue acidosis, NOX2 expression, ROS production, proinflammatory cytokine production, microglia proliferation, leukocyte infiltration, and phagocytic oxidative burst at 3 days post-injury (Li et al., 2021). Tissue pH levels were markedly lower in the first week after SCI. Tissue acidosis was most evident at the injury site, but also extended into proximal regions of the cervical and lumbar cord. Tissue ROS levels and expression of Hv1 were also significantly increased during the first week of injury. In the long term, Hv1 KO mice exhibited significantly improved locomotor function and reduced histopathology. Meanwhile, other groups have reported similar results as well as Hv1’s role in attenuating reactive astrogliosis, and reducing oligodendrocyte apoptosis in SCI models (Li et al., 2020a; Murugan et al., 2020). Overall, these data suggest an important role for Hv1 in regulating extracellular acidosis, NOX2-mediated ROS production, and functional outcome following SCI (Li et al., 2021). Thus, the Hv1 proton channel represents a potential target that may lead to novel therapeutic strategies for TBI and SCI.

However, injured brain and spinal cord are invaded by blood-borne leukocytes expressing high levels of Hv1 and NOX. To further investigate whether peripheral Hv1 contributes to tissue damage after CNS trauma, bone marrow chimeric mice will be needed to examine respective contribution of Hv1 in microglia and peripheral immune cells to neuroinflammation as well as to functional outcomes.

## Status of Drug Discovery

### Hv1 as a Novel Drug Target

Since Hv1 is involved in the regulation of NOX2, it offers a potential advantage as a drug target over NOX2, as NOX2 deficiency completely abolishes ROS production that is beneficial and necessary for supporting the function and viability of cells under non-injury conditions. In contrast, it is postulated that Hv1 deficiency may keep at least 30% of ROS, enough to supply vital cellular functions and maintenance (Seredenina et al., 2015). As an acid extruder that is independent of NOX2, Hv1 is indicated in the treatment of not only CNS injury but also cancer, obesity, diabetes, and allergy (Bare et al., 2020; Kawai et al., 2020; Pang et al., 2020). In addition to the development of Hv1 inhibitors, Hv1 activators might also be of interest for certain types of pathologies such as male infertility and possibly autoimmune disease (Seredenina et al., 2015).

### Proton Channel Blockers

Zn^2+^ and other polyvalent cations have been used for many years as proton current inhibitors. These compete with H^+^ for binding to the external surface, which changes the membrane potential perceived by the channel. However, this inhibition is non-specific, as Zn^2+^ ions are implicated in many other physiological processes. Thus, the usefulness of Zn^2+^ as an Hv1 channel blocker is limited. Hanatoxin, a toxin from the venom of the tarantula *Grammostola spatulate*, is another non-specific blocker of Hv1. This molecule binds to the paddle motif, which is highly conserved among different voltage-gated ion channels (Seredenina et al., 2015).

Some guanidine derivatives have been shown to have the ability to inhibit Hv1 activity. One of these compounds, 2-guanidinobenzimidazole (2GBI) binds the channel’s voltage-sensitive domain only in the open conformation. The binding site is within the proton permeation pathway and faces the cytoplasm (Hong et al., 2013). However, 2GBI is too polar to permeate the cytoplasmic membrane, and thus prevents it from being considered a potential Hv1 inhibitor. A modified version of 2GBI (CIGBI) that makes it more permeable to the cell membrane was later identified to bind Hv1 more efficiently, making a step forward to the development of pharmacological treatments for diseases caused by Hv1 hyperactivity (Hong et al., 2014). Nonetheless, the relative low potency and uncertainty about specificity against Hv1 remains a problem (Pupo and Gonzalez Leon, 2014).

Several proton current inhibitors, such as 4-aminopyridine, amantadine, amiloride, D600, nicardipine, imipramine, DM, chlorpromazine, clozapine, haloperidol, and rimantadine have potentially indirect effects on Hv1 function, presumably by increasing intracellular pH (Shin et al., 2015). In particular, epigallocatechin-3-gallate might interfere with Hv1 channel activity by modifying the lipid bilayer structure (Seredenina et al., 2015; Fernandez et al., 2016). Additionally, a toxic peptide C6 was identified as a high potential Hv1 blocker with specificity and high affinity (Kd = 1 nM) (Tang et al., 2020). Two C6 peptides bind to each dimeric channel on the S3–S4 loop of the VSD, shifting human Hv1 activation to more positive voltages, slowing opening and speeding closure, which consequently diminishes the membrane depolarization (Zhao et al., 2018). The scorpion toxin anti-tumor analgesic peptide (AGAP/W38F) is another novel selective Hv1 channel antagonist. Its binding pocket on the Hv1 channel partly overlaps that of Zn^2+^ with even less pH dependence. Therefore, AGAP/W38F could be a useful probe for exploring the structure–function relationship of the Hv1 channel and may have strong therapeutic potential (Tang et al., 2020).

### Mechanisms and Regulation of Hv1 Channel Activation

Currently, no drug-like molecules are known to activate Hv1. Unsaturated long chain fatty acids such as arachidonic acid (AA) are known to modulate the activities of various ion channels, including Hv1 (Kawanabe and Okamura, 2016). Application of AA, for example, rapidly induced a robust increase in the amplitude of the proton current through Hv1. The application of phospholipase A2 (PLA2), which generates AA from cell membrane phospholipids, stimulated Hv1 activity to a similar extent as the direct application of 20 μM AA, suggesting that endogenous AA may regulate Hv1 channel activity (Kawanabe and Okamura, 2016).

## Conclusion and Perspectives

Ever since the identification of the gene in 2006, there has been growing interest in the Hv1 proton channel and its role in immunity, CNS injury, metabolic disease and cancer. Yet many questions remain unanswered. Though it is widely accepted that Hv1 interacts with NOX2 to regulate the production of ROS, it is not clear which one is upstream. In acute CNS injury, Hv1 deficiency diminishes ROS production, reduces expression of inflammatory cytokines, and ameliorates extracellular acidosis. However, whether this is primarily caused by the residential microglia or infiltrating myeloid cells is still not clear. In the future, the development of cell-specific Hv1 KO mice will be necessary to address the respective contributions of each phagocyte population to the pathogenesis of acidotic and oxidative stress damage following CNS injury.

Structural exploration could not only help with the understanding of the molecular mechanisms of channel activation and proton permeation (Chamberlin et al., 2015; Li et al., 2015; De La Rosa et al., 2018), but also facilitate the discovery of drugs targeting Hv1 (Lee et al., 2018; Jardin et al., 2020). These drug candidates, in turn, will help further our understanding of Hv1 function in health and disease.

## Author Contributions

All authors listed have made a substantial, direct and intellectual contribution to the work, and approved it for publication.

## Conflict of Interest

The authors declare that the research was conducted in the absence of any commercial or financial relationships that could be construed as a potential conflict of interest.
